# Incremental prognostic value of aortic stiffness in addition to myocardial ischemia by cardiac magnetic resonance imaging

**DOI:** 10.1186/s12872-020-01550-w

**Published:** 2020-06-11

**Authors:** Yodying Kaolawanich, Thananya Boonyasirinant

**Affiliations:** Division of Cardiology, Department of Medicine, Faculty of Medicine Siriraj Hospital, Mahidol University, 2 Wanglang Road, Bangkok Noi, Bangkok, 10700 Thailand

**Keywords:** Aortic stiffness, Cardiac magnetic resonance imaging, Myocardial ischemia, Prognosis

## Abstract

**Background:**

Aortic stiffness is an independent predictor of cardiovascular (CV) events and mortality. However, no data exists for the prognosis of combined aortic stiffness and myocardial ischemia. Using cardiac magnetic resonance (CMR) imaging, we assessed the association of aortic stiffness by pulse wave velocity (PWV), myocardial ischemia, and CV events in patients with known or suspected coronary artery disease (CAD).

**Methods:**

Velocity-encoded CMR was performed in 520 patients who had undergone adenosine stress CMR. The PWV was determined between the mid-ascending and mid-descending thoracic aorta. Patients were divided into 4 groups by PWV (higher or lower PWV) and myocardial ischemia (positive or negative ischemia). Combined CV events including mortality, acute coronary syndrome, heart failure, coronary revascularization, and stroke were analyzed among the 4 groups.

**Results:**

The median follow-up period was 46.5 months, and the median PWV was 10.54 m/sec. Myocardial ischemia was positive in 199 patients (38.3%). The group with a higher PWV and positive ischemia had the most CV events (hazard ratio 8.94, *p* <  0.001). The group with a higher PWV and negative ischemia also was significantly associated with CV events (HR 2.19, *p* = 0.02). Groups with a lower PWV-positive ischemia and a higher PWV-negative ischemia showed no difference in terms of CV events (HR 0.60, *p* = 0.08). Patients with myocardial ischemia who had higher PWV demonstrated significantly higher event rates than those who had lower PWV (HR 2.41, *p* <  0.001). Multivariate analysis demonstrated that myocardial ischemia and PWV were independent predictors for combined CV events (HR 2.71, *p* <  0.001 and HR 2.42, *p* <  0.001, respectively).

**Conclusions:**

Stress perfusion CMR provided prognostic utility in patients with known or suspected CAD. Adding aortic stiffness to stress perfusion CMR could improve risk assessment and prediction for future CV events.

## Background

Aortic stiffness is one of the earliest detectable indicators of adverse structural and functional changes in the vessel wall [1]. Several factors or diseases affect aortic stiffness, including increasing age [2–4], smoking [5], obesity [6], hypertension [7, 8], dyslipidemia [9, 10], impaired glucose tolerance [11], diabetes mellitus [11], metabolic syndrome [11], hyperhomocysteinemia [12], and high C-reactive protein levels [13, 14]. Aortic stiffness has been proven to be an independent predictor when evaluated with traditional risk factors for cardiovascular morbidity and mortality [15, 16].

Measurement of aortic stiffness can be performed by several methods, such as carotid-femoral pulse wave velocity (PWV) using a tonometer, computed tomography, or cardiac magnetic resonance (CMR) imaging. Each method has its advantages and disadvantages. Although carotid-femoral PWV is inexpensive and available, CMR is often preferred for several reasons. The advantages of PWV using CMR include the provision of cross-sectional images covering the desired length of the aorta, a high spatial resolution, direct measurement of the aorta length without geometric assumptions of the distance (in contrast to tonometer), a lack of ionizing radiation, and the ability to evaluate other aspects of the aorta (such as aortic wall strain and deformation) [17, 18]. Furthermore, CMR-based PWV measurements have been well validated (compared with invasive pressure recordings), and they have a high reproducibility [19].

PWV assessment can be integrated in a comprehensive CMR study for coronary artery disease (CAD) evaluation. Adenosine stress CMR, a noninvasive test for the highly sensitive and specific diagnosis of CAD [20], provides information regarding cardiac function, perfusion, and myocardial scarring. Adenosine stress CMR also offers strong evidence for the prognosis of newly developed cardiovascular events, including mortality in patients with known or suspected CAD [21]. Nevertheless, no data exists for the prognosis of combined aortic stiffness and myocardial ischemia.

This study aimed to determine whether aortic stiffness by CMR-based PWV can add the prognostic value to myocardial ischemia for the prediction of future cardiovascular events.

## Methods

This retrospective, single-center study was approved by the center’s institutional ethics committee. A total of 520 patients referred for adenosine stress CMR as part of the diagnosis and risk stratification of CAD were consecutively enrolled at Siriraj Hospital (Mahidol University, Bangkok, Thailand) between October 2010 and February 2014. The inclusion criteria were males or females aged 18 or older who underwent adenosine stress CMR and aortic PWV, with a follow-up period of at least 12 months following the CMR examination. Patients with (1) an incomplete CMR examination or (2) diseases of the aorta which involved PWV measurement (e.g., an aortic aneurysm) were excluded.

Detailed medical history and vital signs including blood pressure (BP) data were recorded from medical records. Further, BP was measured in a supine position by automated BP devices using appropriate cuff size, on the same day of CMR examination (approximately 10 min before scanning). The BP values were averaged from two measurements for each patient.

The follow-up data were collected from clinical visits, medical records, and scripted telephone interviews. Demographic data, cardiovascular risk factors, adenosine stress CMR, and PWV results were also included. Based on their PWV and ischemia results, patients were divided into 4 groups as higher or lower PWV (using mean or median), and positive or negative ischemia.

The primary outcome was to assess the combined cardiovascular outcomes (all-cause mortality, acute coronary syndrome [ACS], de novo or decompensated heart failure, coronary revascularization, and stroke) of the patients in each group. The secondary outcomes were identified as the results of each primary outcome and cardiovascular mortality. The predictors of the combined cardiovascular outcomes were analyzed. Since adenosine stress CMR results may influence decisions regarding revascularization and lead to periprocedural events or death, coronary revascularizations which occurred within 6 months of the CMR, or periprocedural events that occurred in the same admission, were not included for analysis.

### Magnetic resonance imaging scanning

The CMR study was performed using a 1.5 T Philips Achieva XR scanner (Philips Medical Systems, Best, The Netherlands). After a scout image to locate the cardiac axis, an electrocardiogram (ECG)-triggered, breath-hold, black blood, single-shot sequence was acquired in the axial orientation for 30 slides; it covered the whole heart and thoracic aorta. The scanning parameters were echo time (TE) 24 milliseconds (ms); repetitive time (TR) 1400 ms; refocusing flip angle 90^°^; field of view (FOV) in x axis 240–360 mm; FOV in y axis 250–300 mm; slide thickness 8 mm; acquisition voxel size 1.75 × 1.75 mm; and reconstructed voxel size 0.64 × 0.64 mm.

The myocardial first-pass perfusion study was assessed immediately after an injection of 0.05 mmol/kg of gadolinium contrast agent (Magnevist, Bayer Schering Pharma, Berlin, Germany) beginning at 3-min of adenosine 0.56 mg/kg infusion. The 3 short-axis slices of apical, mid, and basal left ventricular levels were acquired using an ECG-triggered, steady-state free precession, inversion-recovery, single-shot, turbo gradient-echo sequence. The image parameters were TE 1.32 ms; TR 2.6 ms; refocusing flip angle 50^°^; slide thickness 8 mm; FOV 270 mm; reconstructed FOV 320 mm; typical matrix size 2.55 × 2.6 mm; and reconstructed spatial resolution 1.3 × 1.3 mm. Continuous ECG monitoring was performed, and 1-min-interval BP and oxygen saturation monitoring were measured.

The PWV image was acquired during the waiting period between the stress and viability studies, approximately 10 min after adenosine injection. The image was determined with the free-breathing, velocity encoded CMR (VE-CMR) technique as the through-plane flow in the mid-ascending and mid-descending thoracic aorta at the level of the pulmonary trunk. The imaging parameters were retrospective ECG-triggered; TE 3.1 ms; TR 5.3 ms; refocusing flip angle 12^°^; FOV in x axis 250 mm; FOV in y axis 210 mm; slide thickness 8 mm; typical matrix size 2.0 × 2.0 mm; reconstructed spatial resolution 1.12 × 1.12 mm; temporal resolution 10–20 ms; and velocity encoding 170 cm/s.

### Adenosine stress CMR analysis

Sixteen myocardial segments were defined for perfusion analysis (in accordance with the standard recommendation of the American Heart Association), excluding the true apex [22]. A myocardial perfusion defect was defined as positive when a perfusion delay persisted for at least 5 consecutive phases in at least 1 segment during the peak myocardial enhancement. The results were assessed by two experienced readers, and in cases of disagreement, a third experienced reader was consulted.

### PWV analysis

Dedicated cardiovascular imaging software was applied for the PWV analysis. The contours of the mid-ascending and mid-descending thoracic aorta were manually drawn to achieve the flow (m/sec) at those 2 locations throughout all phases of the cardiac cycle. The corresponding flow-time curve was generated. The arrival time of the pulse wave was measured as the point of interception of the linear extrapolation of the baseline and the steep early systolic stage. The aortic path length was determined by multiplanar reconstruction of axial half-Fourier acquisition from the steady stage image. As to the reconstructed sagittal view, the path length was depicted as the centerline from the levels of the mid-ascending aorta to the mid-descending thoracic aorta, corresponding to the same level obtained in the VE-CMR.

The PWV between the mid-ascending and mid-descending thoracic aorta was calculated using the following formula:

$$ \mathrm{PWV}=\Delta\ \mathrm{x}/\Delta\ \mathrm{T}\ \left(\mathrm{m}/\sec \right) $$

**Fig. 1:**
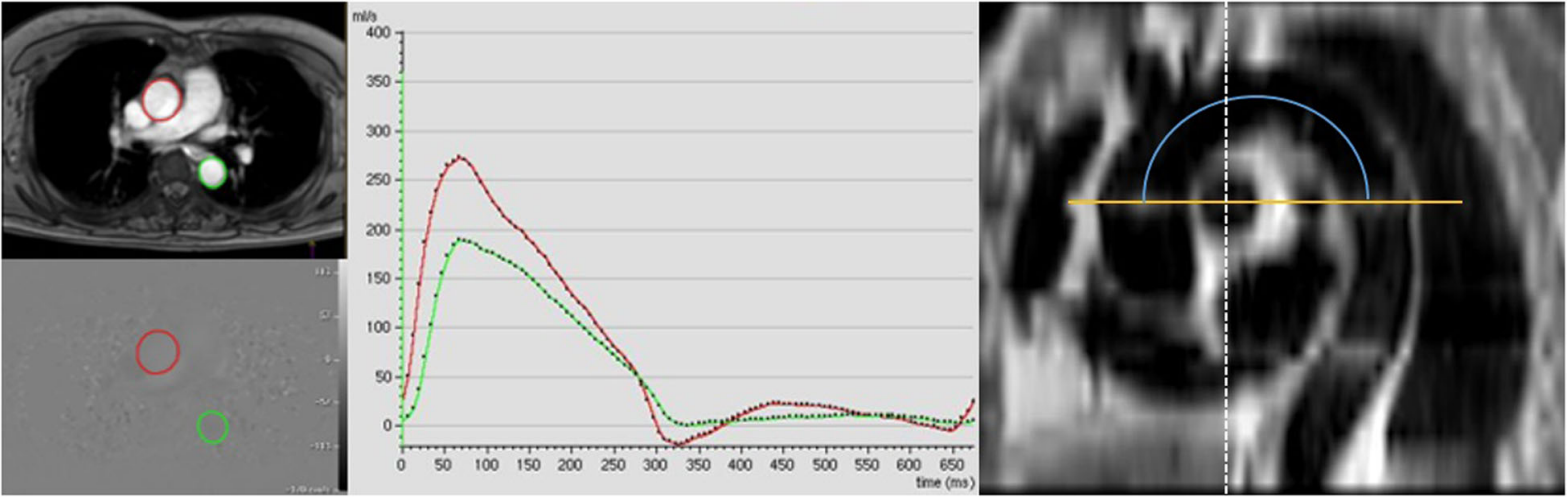
Measurement of time delay between pulse waves and aortic path length. Left: Through-plane velocity-encoded cardiac magnetic resonance at the mid-ascending (red circles) and mid-descending thoracic aorta (green circles). Middle: Corresponding flow measurement at the mid-ascending (red line) and mid-descending thoracic aorta (green line). Right: The measurement of aortic path length using a multiplanar reconstructed oblique sagittal view

When Δ x reflected the length of the aortic path between the mid-ascending and mid-descending thoracic aorta and Δ T represented the time delay between the arrival of the foot of pulse wave at those two corresponding levels (Fig. 1). The analyses of adenosine stress tests and PWV were performed independently.

### Intra- and inter-observer reliability

To assess the intra- and inter-observer reliability of the PWV measurements by CMR, 50 patients were randomly selected in order to measure the variability of the first observer 4 weeks after the initial analysis, and the variability of the second independent observer, who was blinded to the initial results.

### Statistical analysis

Statistical analyses were performed using IBM SPSS Statistics for Windows, version 20.0 (IBM Corp., Armonk, NY, USA). Normally distributed continuous data were expressed as mean and standard deviation (SD). Non-normally distributed continuous data were expressed as median and interquartile range. Categorical data were expressed as number and percentages. Normally distributed continuous data of multiple (> 2) groups were compared using one-way analysis of variance (ANOVA). Non-normally distributed continuous data of multiple (> 2) groups were compared using Kruskal-Wallis test. Categorical data were compared by Chi-squared or Fisher’s exact test.

Cox regression was used for unadjusted and adjusted hazard ratios. Kaplan-Meier event curves for the 4 groups of patients were constructed for the combined cardiovascular outcomes of all-cause mortality, ACS, de novo or decompensated heart failure, coronary revascularization, and stroke.

To analyze the predictive value of PWV and myocardial ischemia for the combined cardiovascular outcomes, a Cox-regression analysis was performed for the assessment of univariate predictors from baseline characteristics, medications, and CMR parameters. Variables with *p*-value < 0.1 on univariate analysis were entered into a multivariate analysis. Multivariable analysis was performed to determine independent predictors. The intra- and inter-observer reliabilities of PWV measurements were analyzed using the intraclass correlation coefficient.

The hazard ratios (HRs) and 95% confidence intervals (CIs) for the various outcomes were calculated; a *p*-value < 0.05 was considered as statistically significant.

## Results

Comprised of 259 males and 261 females, a total of 520 patients were consecutively enrolled. Their mean age was 68.9 ± 10.6 years, and their baseline characteristics and CMR parameters are summarized in Table 1. The average left ventricle ejection fraction (LVEF) was 66.7% ± 14.7%. The median PWV (interquartile range) was 10.54 (7.86–13.89) m/sec. The median follow-up time (interquartile range) was 46.5 (33.0–67.1) months. Using the median PWV as the cut-off value, patients were further classified according to their PWV results into those with higher PWVs (≥ 10.54 m/sec) and lower PWVs (< 10.54 m/sec). In all, 108 patients (20.8%) had a previous history of CAD. The adenosine stress CMR was positive in 199 cases (38.3%). Patients were stratified into 4 groups (group 1 as lower PWV-negative ischemia, group 2 as lower PWV-positive ischemia, group 3 as higher PWV-negative ischemia, and group 4 as higher PWV-positive ischemia). Group 1 (lower PWV-negative ischemia) was the reference group for the outcome assessment, though a specific comparison was mentioned.

**Table 1:** Baseline characteristics and CMR parameters of all patients

	Group 1 Lower PWV Negative ischemia (*n* = 169)	Group 2 Lower PWV Positive ischemia (*n* = 91)	Group 3 Higher PWV Negative ischemia (*n* = 152)	Group 4 Higher PWV Positive ischemia (*n* = 108)	Total	*p*-value
Age, years	64.6 ± 11.7	67.2 ± 9.9	73.1 ± 8.6	70.9 ± 9.3	68.9 ± 10.6	**< 0.001**
Female	91 (53.8)	30 (33.0)	93 (61.2)	47 (43.5)	261 (50.2)	**< 0.001**
BMI, kg/sqm	26.9 ± 4.6	27.1 ± 4.6	26.9 ± 4.5	25.4 ± 3.6	26.7 ± 4.4	**0.01**
Previous history						
Hypertension	134 (79.3)	76 (83.5)	142 (93.4)	96 (88.9)	448 (86.2)	**0.002**
Diabetes mellitus	75 (44.4)	48 (52.7)	103 (67.8)	65 (60.2)	291 (56.0)	**< 0.001**
Dyslipidemia	114 (67.5)	71 (78.0)	114 (75.0)	80 (74.1)	379 (72.9)	0.25
Known CAD	12 (7.1)	29 (31.9)	24 (15.8)	43 (39.8)	108 (20.8)	**< 0.001**
Stroke	7 (4.1)	4 (4.4)	5 (3.3)	6 (5.6)	22 (4.2)	0.83
Current smoking	14 (8.3)	27 (29.7)	7 (4.6)	20 (18.5)	68 (13.1)	**< 0.001**
Medications						
Aspirin	67 (39.6)	55 (60.4)	66 (43.4)	70 (64.8)	258 (49.6)	**< 0.001**
Beta blocker	75 (44.4)	48 (52.7)	78 (51.3)	56 (51.9)	257 (49.4)	0.46
Calcium channel blocker	50 (29.6)	26 (28.6)	55 (36.2)	33 (30.6)	164 (31.5)	0.53
ACEI or ARB	62 (36.7)	40 (44.0)	77 (50.7)	57 (52.8)	236 (45.4)	**0.03**
Statin	83 (49.1)	49 (53.8)	80 (52.6)	74 (68.5)	286 (55.0)	**0.01**
Parameter						
Systolic BP, mmHg	131.8 ± 18.4	132.3 ± 18.0	139.5 ± 19.2	144.6 ± 21.5	136.8 ± 19.9	**< 0.001**
Diastolic BP, mmHg	74.4 ± 10.9	72.1 ± 12.4	72.1 ± 12.6	72.6 ± 12.6	72.9 ± 12.0	0.282
Pulse pressure, mmHg	57.3 ± 15.6	60.2 ± 16.2	67.5 ± 17.2	71.7 ± 20.7	63.8 ± 18.2	**< 0.001**
Heart rate, bpm	76.9 ± 13.4	75.2 ± 12.4	77.5 ± 14.8	77.5 ± 13.8	76.9 ± 13.7	0.54
LVEF, %	68.2 ± 12.5	61.1 ± 16.2	72.9 ± 9.5	60.6 ± 18.1	66.7 ± 14.7	**< 0.001**
PWV, m/sec	7.73 (6.64–8.94)	8.46 (7.08–9.43)	13.99 (12.35–17.59)	13.44 (11.57–16.31)	10.54 (7.86–13.89)	**< 0.001**

Values are n (%), mean ± SD, or median (interquartile range). Bold values are statistically significant

*ACEI* Angiotensin-converting enzyme inhibitors, *ARB* Angiotensin-receptor blockers, *BMI* Body mass index, *CAD* Coronary artery disease, *BP* Blood pressure, *LVEF* Left ventricular ejection fraction, *PWV* Pulse wave velocity, *SD* Standard deviation

### Combined cardiovascular outcomes

The cumulative event rates for the composite outcomes of all-cause mortality, ACS, de novo or decompensated heart failure, coronary revascularization, and stroke in each group are presented in Table 2. The overall combined endpoints were 116 events (22.3%).

**Table 2:** Cardiovascular outcomes of all patients

	Group 1 Lower PWV-Negative ischemia	Group 2 Lower PWV-Positive ischemia				Group 3 Higher PWV-Negative ischemia				Group 4 Higher PWV-Positive ischemia			
	Event Rate (%)	Event Rate (%)	Unadjusted HR, *p*-value	Adjusted HR model 1, *p*-value	Adjusted HR model 2, p-value	Event Rate (%)	Unadjusted HR, p-value	Adjusted HR model 1, *p*-value	Adjusted HR model 2, *p*-value	Event Rate (%)	Unadjusted HR, *p*-value	Adjusted HR model 1, *p*-value	Adjusted HR model 2, *p*-value
All-cause Mortality	6 (3.6)	10 (11.0)	3.17, **0.03**	3.13, **0.03**	3.54, **0.02**	3 (2.0)	0.61, 0.49	0.59, 0.47	0.62, 0.51	9 (8.3)	2.77, 0.05	3.01, **0.04**	2.85, 0.08
CV Mortality	1 (0.6)	5 (5.5)	9.52, **0.04**	8.53, 0.05	5.05, 0.17	0	N/A	N/A	N/A	4 (3.7)	7.35, 0.07	7.93, 0.07	4.20, 0.25
ACS	3 (1.8)	8 (8.8)	5.18, **0.02**	5.49, **0.01**	4.80, **0.03**	8 (5.3)	3.39, 0.07	3.19, 0.09	2.95, 0.12	22 (20.4)	15.14, **< 0.001**	15.41, **< 0.001**	12.09, **< 0.001**
Heart failure	5 (3.0)	12 (13.2)	4.78, **0.003**	4.72, **0.004**	3.60, **0.02**	12 (7.9)	2.71, 0.06	2.47, 0.09	2.91, 0.06	25 (23.1)	10.03, **< 0.001**	11.18, **< 0.001**	8.06, **< 0.001**
Revascularization	0	6 (6.6)	N/A	N/A	N/A	3 (2.0)	N/A	N/A	N/A	20 (18.5)	N/A	N/A	N/A
Stroke	1 (0.6)	2 (2.2)	3.89, 0.30	3.55, 0.13	3.00, 0.38	5 (3.3)	6.42, 0.09	5.42, 0.13	6.09, 0.11	6 (5.6)	11.94, **0.02**	10.31, **0.03**	7.98, 0.07
Combined CV Outcomes	14 (8.3)	25 (27.5)	3.67, **< 0.001**	3.66, **< 0.001**	2.68, **0.005**	24 (15.8)	2.19, **0.02**	2.24, **0.02**	2.29, **0.02**	53 (49.1)	8.94, **< 0.001**	9.86, **< 0.001**	6.67, **< 0.001**

Bold values are statistically significant

The group 1 (lower PWV with negative ischemia) is the reference group

Model 1 is adjusted for age, gender, and systolcic blood pressure

Model 2 is adjusted for age, gender, systolic blood pressure, BMI, hypertension, diabetes mellitus, known CAD, current smoking, and LVEF

*ACS* Acute coronary syndrome, *BMI* Body mass index, *CAD* Coronary artery disease, *CV* Cardiovascular, *HR* Hazard ratio, *LVEF* Left ventricular ejection fraction, *N/A* Not available, *PWV* Pulse wave velocity

Using the group 1 (lower PWV-negative ischemia) as the reference group, the group 4 (higher PWV-positive ischemia) had the significantly worst outcomes for the combined endpoints (HR 8.94, 95% CI 4.95–16.14, *p* <  0.001). The group 2 (lower PWV-positive ischemia) and the group 3 (higher PWV-negative ischemia) also showed significantly increased the combined endpoints (HR 3.67, 95% CI 1.91–7.06, *p* <  0.001, and HR 2.19, 95% CI 1.13–4.20, *p* = 0.02, respectively). Remarkably, there were no significant differences between the group 2 and 3 in terms of the event rates (using the group 2 as the reference: HR 0.60, 95% CI 0.34–1.05, *p* = 0.08). Additionally, patients with myocardial ischemia (groups 2 and 4) who had higher PWV (group 4) demonstrated significantly higher event rates than those who had lower PWV (group 2) (using group 2 as the reference: HR 2.41, 95% CI 1.49–3.88, *p* <  0.001).

**Fig. 2:**
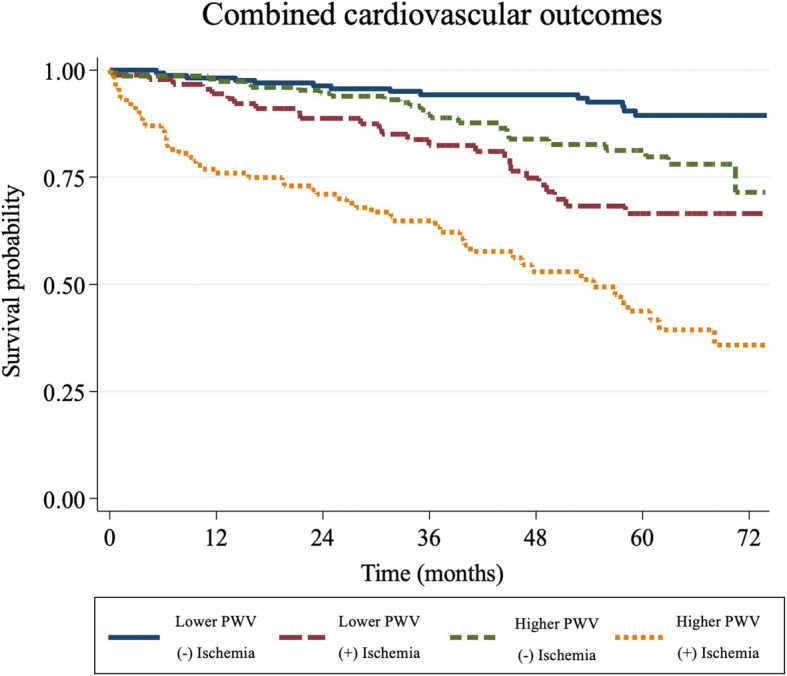
Kaplan–Meier event curves for combined cardiovascular outcomes. PWV = pulse wave velocity

After adjusting for traditional risk factors and LVEF, the group 2–4 maintained significant association with combined cardiovascular outcomes (Table 2). Kaplan–Meier survival curves for combined cardiovascular outcomes are shown in Fig. 2.

### All-cause and cardiovascular mortality

During the follow-up period, 28 total deaths including 10 cardiovascular deaths were recorded. Cardiovascular disease was the leading cause of mortality (35.7%), followed by infection (28.6%) and cancer (21.4%). The groups 2 and 4 with positive ischemia by adenosine stress CMR had the highest mortality rate (Table 2).

### ACS

**Fig. 3:**
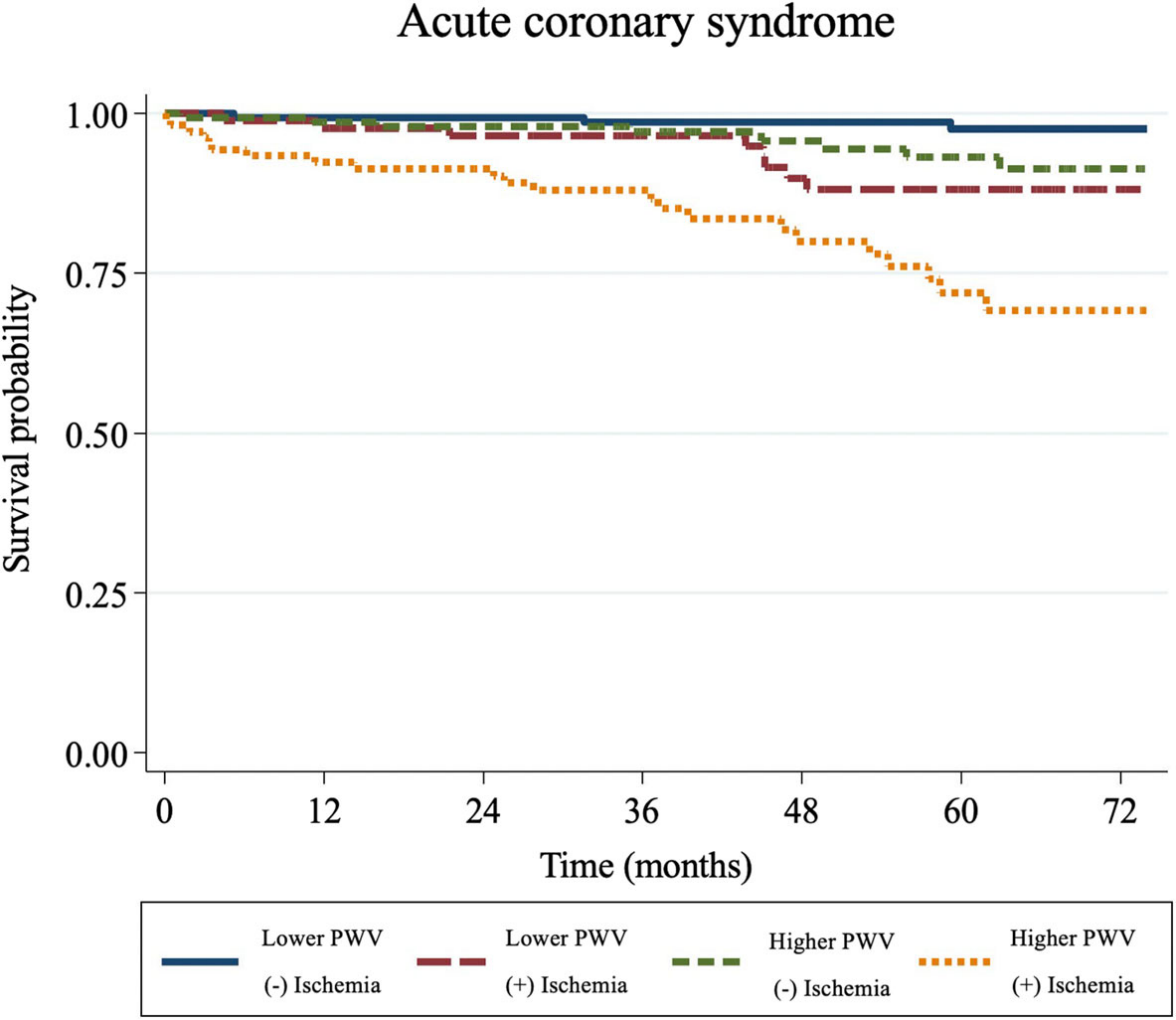
Kaplan–Meier event curves for acute coronary syndrome. PWV = pulse wave velocity

ACS was defined as the composite of unstable angina, non-ST-segment elevation myocardial infarction (NSTEMI), and ST-segment elevation myocardial infarction (STEMI). There were 41 cases of ACS (unstable angina = 10, NSTEMI = 25, and STEMI = 6), 30 (73%) of which were in the higher PWV groups (group 3 and 4). The Kaplan–Meier event curves for the ACS cases in each group are illustrated in Fig. 3.

### Heart failure

**Fig. 4:**
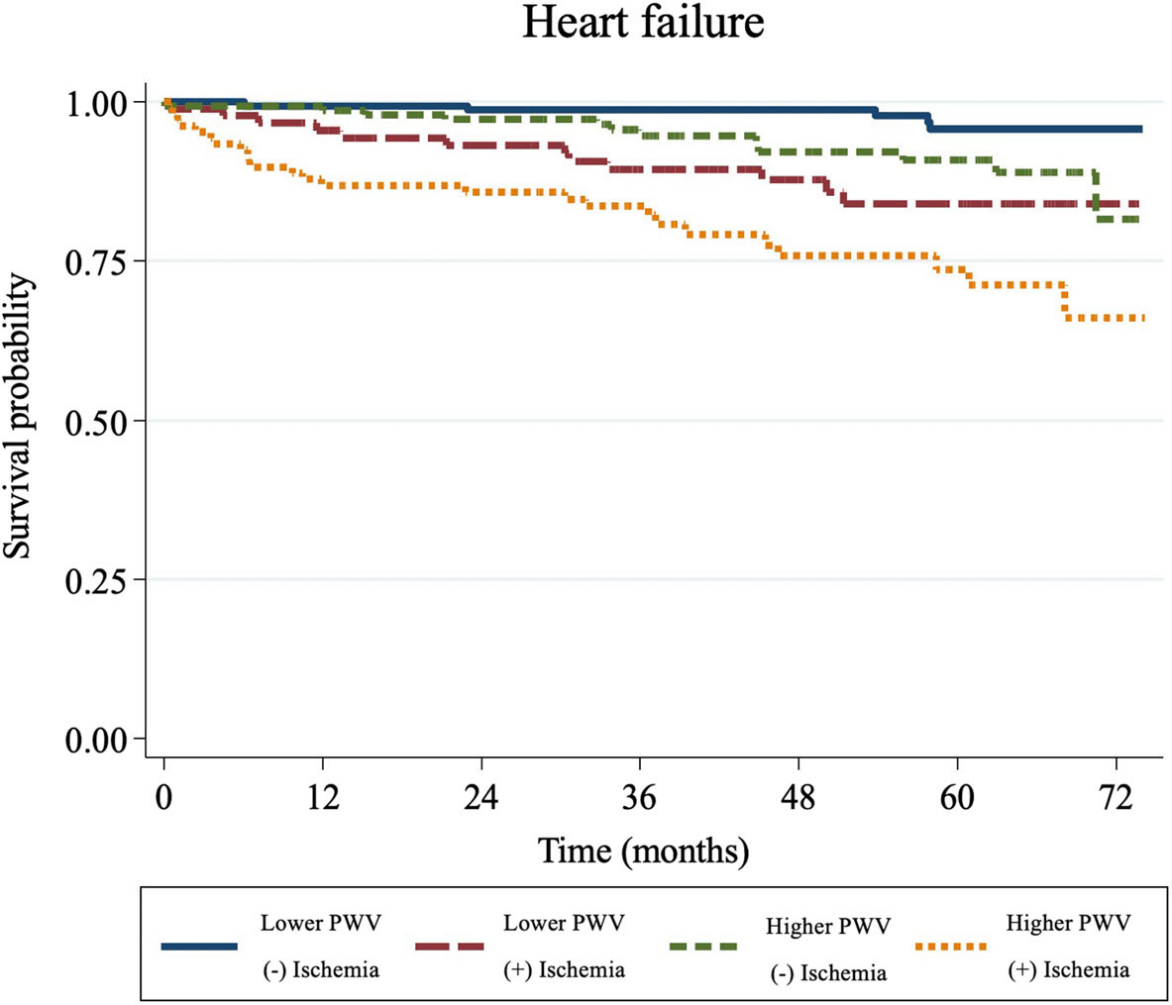
Kaplan–Meier event curves for de novo or decompensated heart failure. PWV = pulse wave velocity

The leading cause of cardiovascular death was de novo or decompensated heart failure; of the 54 events recorded, 43 (80%) required hospital admission. The higher PWV groups (group 3 and 4) were associated with an increased rate of de novo or decompensated heart failure than the groups with a lower PWV (group 1 and 2) (HR 2.49, 95% CI 1.40–4.42, *p* = 0.002). The Kaplan–Meier curves for the heart failure events are presented in Fig. 4.

### Coronary revascularization

Coronary revascularizations occurring at least 6 months after the CMR examination were included in the combined endpoint. There were 29 coronary revascularizations, including 20 with percutaneous coronary intervention and 9 requiring coronary bypass grafting. Twenty-three of those revascularizations were in the higher-PWV groups (group 3 and 4).

### Stroke

There were 14 strokes during the follow-up period. Most stroke events (11 from 14) was in the higher PWV group (3 and 4). All strokes were ischemic in origin, and most patients recorded a good recovery without major morbidity.

### Univariate and multivariate analyses

The univariate and multivariable analyses to predict the combined cardiovascular outcomes are shown in Table 3. The univariate analysis revealed age, body mass index, known CAD, LVEF, myocardial ischemia, and PWV as predictors, while the multivariate analysis revealed only LVEF (HR 1.26, 95% CI 1.12–1.42, *p* <  0.001 [per 10% decrement]), myocardial ischemia (HR 2.71, 95% CI 1.75–4.21, *p* <  0.001), and PWV (HR 2.42, 95% CI 1.64–3.57, *p* <  0.001) as independently associated with combined cardiovascular events.

**Table 3:** Univariate and multivariate analysis of variables for combined cardiovascular outcomes

Variables	Combined cardiovascular outcomes			
	Univariate analysis		Multivariate analysis	
	HR (95% CI)	*p*-value	HR (95% CI)	*p*-value
Age (per 10 years increment)	1.17 (0.99–1.41)	**0.08**	1.10 (0.91–1.33)	0.34
Male	1.16 (0.81–1.67)	0.42	–	–
Body mass index	0.96 (0.91–0.99)	**0.04**	0.98 (0.94–1.03)	0.50
Hypertension	1.36 (0.76–2.42)	0.29	–	–
Diabetes mellitus	1.24 (0.86–1.80)	0.25	–	–
Dyslipidemia	1.18 (0.77–1.82)	0.45	–	–
Known coronary artery disease	2.35 (1.59–3.48)	**< 0.001**	1.48 (0.98–2.24)	0.06
Stroke	1.19 (0.52–2.71)	0.68	–	–
SBP (per 10 mmHg increment)	1.01 (0.92–1.11)	0.77	–	–
LVEF (per 10% decrement)	1.37 (1.24–1.52)	**< 0.001**	1.26 (1.12–1.42)	**< 0.001**
Myocardial ischemia	4.01 (2.72–5.91)	**< 0.001**	2.71 (1.75–4.21)	**< 0.001**
PWV ≥ 10.54 m/sec	2.42 (1.64–3.56)	**< 0.001**	2.42 (1.64–3.57)	**< 0.001**

Bold values are statistically significant

*CI* Confidence interval, *HR* Hazard ratio, *LVEF* Left ventricular ejection fraction, *PWV* Pulse wave velocity, *SBP* Systolic blood pressure

### Intra- and inter-observer reliability

**Fig. 5:**
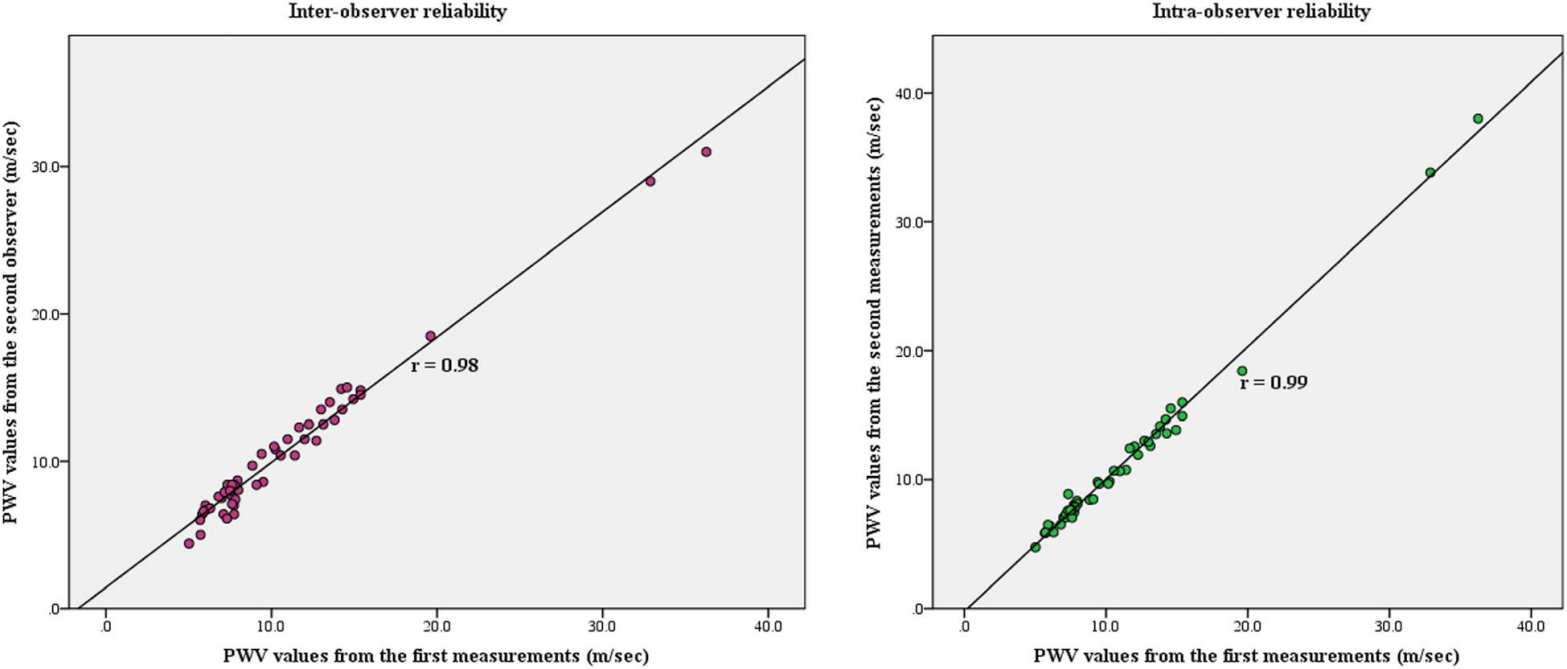
Inter- (left) and intra-observer (right) reliability of PWV measurements. PWV = pulse wave velocity

Excellent intra- and inter-observer reliabilities were demonstrated for PWV measurements by CMR. For the 50 randomly-selected patients, the mean PWV ± SD values were 10.72 ± 5.95 m/sec and 10.79 ± 6.13 m/sec (r = 0.99; *p* <  0.001) for the first observer in the initial analysis and 4 weeks later, respectively, and 10.55 ± 5.12 m/sec (r = 0.98; *p* <  0.001) for the second observer in the initial analysis (Fig. 5).

## Discussion

The three main findings of this study were 1) aortic stiffness measured by CMR independently predicted composite cardiovascular events in patients with known or suspected CAD underwent adenosine stress test; 2) the presence of inducible myocardial ischemia was a powerful predictor for cardiovascular events; and 3) the combination of aortic stiffness and myocardial ischemia provided significant improvement of prognostic predictions.

### Aortic stiffness

Arterial stiffness refers to alterations of medial properties leading to reduced distensibility of the arterial wall. Many factors and diseases influence arterial stiffness, including aging [2–4], hypertension [7, 8], diabetes mellitus [11], dyslipidemia [9, 10], and smoking [5].

Several functional and structural changes contribute to arterial stiffness, for instance, high BP, impaired smooth muscle function, impaired endothelium-dependent dilation, increased collagen content expression, and decreased elastin content. Furthermore, numerous potentials signaling events contribute to age- and disease-related arterial stiffness, such as oxidative stress, inflammation, and decreased expression of endothelial nitric oxide synthase activity. Increased aortic stiffness has been established in various cardiovascular diseases and metabolic abnormalities.

Carotid-femoral PWV using a tonometer is generally accepted as a simple, noninvasive, and inexpensive method to measure arterial stiffness. This technique is the measure used in most clinical studies and is a strong predictor of cardiovascular events [6, 15, 16]. However, it has some limitations. This method requires the assumed measurement of the aortic distance from the carotid to femoral arteries. Most studies measure this distance with tape over the surface of the body, leading to an overestimation of the real distance traveled by the pulse wave [6, 15, 16].

PWV measurement using CMR is one of the preferred methods for evaluation of arterial stiffness, giving high spatial resolution without ionizing radiation. This technique can assess PWV accurately across any segment of aorta, but the level of the mid-ascending and mid-descending aorta was chosen due to the corresponding location of the heart in CMR examination. Moreover, CMR can measure the distance of the aorta without geometrical assumptions, unlike carotid-femoral PWV using a tonometry. The PWV measured by CMR in our study demonstrated excellent images with significantly high reliability comparable to the previous studies [19].

### Relationship between aortic stiffness and myocardial ischemia

The pathophysiology pathway of increased arterial stiffness and myocardial ischemia is a complex issue. One of the explanations could be the translation of the arterial wave reflection progressively from diastole into late systole [23].

Arterial stiffness causes an early arrival of wave reflections in systole instead of diastole and, thus, increases the systolic afterload and reduces the diastolic coronary perfusion pressure. As a result of this pathophysiological changes, arterial stiffness may cause an ischemic heart injury through a reduction in the subendocardial oxygen supply and increase in oxygen demand. However, a recent meta-analysis demonstrated that the arterial reflection times of most subjects were well within systole rather than diastole [24]. This meta-analysis highlighted the concept of the mechanism of BP change with aging. Thus, it appears that arterial stiffness contributes to myocardial ischemia through the loss of arterial compliance itself rather than a change in the reflecting time. Nevertheless, this issue remains a topic of debate and requires further researches.

With regard to CAD, aortic stiffness is associated with a broad range of patients, from apparently healthy subjects to those with cardiovascular risks and stable CAD, to post-coronary intervention [25–28]. One clinical study demonstrated that increased aortic stiffness using brachial-ankle PWV measured by volume-plethysmography was an independent predictor of 3-year cardiovascular event-free survival in patients with established CAD [28]. However, no data on the prognosis using CMR in patients with known or suspected CAD combined with aortic stiffness and myocardial ischemia has been presented.

Previous studies have shown that myocardial ischemia is one of the most important predictors of future hard cardiac events [29, 30]. This statement was supported by our results. We found that myocardial ischemia by CMR was the strongest predictor of combined cardiovascular outcomes. In addition, the result also demonstrated almost 2.5-times increased in composite endpoints in patients who had higher PWV with positive ischemia compare to the group of lower PWV-positive ischemia. Therefore, the potential role of aortic stiffness combination with myocardial ischemia to improve the risk stratification beyond myocardial ischemia alone was highlighted in this study.

### Using CMR to comprehensive assessment of CAD and aortic stiffness

The utility of CMR in the evaluation of CAD is increasingly being recognized. In particular, vasodilator stress perfusion CMR and viability assessment by delayed enhancement technique. The accuracy of the adenosine stress CMR for diagnosis of CAD is substantially high with sensitivity and specificity of 89 and 87%, respectively [21].

Aortic stiffness using PWV can be performed as a part of a comprehensive stress perfusion CMR protocol. In our study, PWV was measured during the waiting period between the stress and viability studies; the non-breath-hold technique proved convenient for patients. The PWV images was acquired after adenosine injection approximately 10 min. Even if adenosine may affect the arterial compliance, given its very rapid half-life (< 10 s), this effect did not alter PWV measurement in our study.

According to the primary study objective, aortic stiffness assessed by CMR was determined as a potential risk marker for cardiovascular disease with high accuracy and reproducibility.

### Limitations

There were some study limitations. Firstly, this study was retrospective in its methodology. Some confounding factors cannot be totally eliminated due to the nature of retrospective study. In addition, no standard, cut-off level was currently used for the PWV quantification. Finally, variations in heart rates may have resulted in slightly different velocity waveforms between cardiac cycles, resulting in errors in the PWV measurements. However, a prior validation study of PWV assessed by CMR determined agreement between invasive intra-aortic pressure measurements [19].

### Clinical application

The addition of aortic stiffness to myocardial ischemia results improves the prognostic prediction of cardiovascular events. The combination of aortic stiffness and adenosine stress test results might become an integral part of clinical risk stratification and the monitoring of therapy.

### Perspectives

Aortic stiffness can be detected long before the clinical appearance of cardiovascular diseases. From our results, measurement of aortic stiffness can be performed along with an assessment of myocardial ischemia, thereby improving cardiovascular risk stratification.

There has been some evidence that has suggested that antihypertensive drugs are beneficial in reducing aortic stiffness and thus might modify cardiovascular events beyond a decrease in BP [31]. This aspect requires further investigation.

## Conclusions

This retrospective study demonstrated that the addition of aortic stiffness to myocardial ischemia could improve the prognostic prediction of CV events. The combination of aortic stiffness and adenosine stress test might become an integral component for risk stratification in CAD.

## Data Availability

The datasets used and/or analyzed during the current study are available from the corresponding author on reasonable request.

## Abbreviations

ACS: Acute coronary syndrome
BP: Blood pressure
CAD: Coronary artery disease
CI: Confidence interval
CMR: Cardiac magnetic resonance imaging
CV: Cardiovascular
ECG: Electrocardiography
FOV: Field of view
HR: Hazards ratio
LVEF: Left ventricular ejection fraction
NSTEMI: Non-ST-segment elevation myocardial infarction
PWV: Pulse wave velocity
SD: Standard deviation
STEMI: ST-segment elevation myocardial infarction
T: Tesla
TE: Echo time
TR: Repetitive time
VE-CMR: Velocity-encoded cardiac magnetic resonance imaging
